# Asthma Control Status Is Associated with Migraine Symptoms in Patients Experiencing Attacks of Both Diseases

**DOI:** 10.31662/jmaj.2025-0123

**Published:** 2025-09-05

**Authors:** Takehito Kobayashi, Takao Atsumi, Yotaro Takaku, Tomoko Suzuki, Naotoshi Tamura, Keiji Yamamoto, Makoto Nagata, Nobuo Araki

**Affiliations:** 1 Allergy Center, Saitama Medical University, Moroyama, Japan; 2 Department of Respiratory Medicine, Saitama Medical University, Moroyama, Japan; 3 General Internal Medicine, Saitama Medical University, Moroyama, Japan; 4 General Internal Medicine, Saitama Prefectural Cardiovascular and Respiratory Center, Saitama, Japan; 5 Department of Neurology, Saitama Medical University, Moroyama, Japan

**Keywords:** bronchial asthma, migraine, comorbidity

## Abstract

**Introduction:**

Numerous reports have suggested a relationship between asthma and migraine. We have encountered patients who presented with migraine attacks in association with asthma attacks.

**Methods:**

In this study, we retrospectively evaluated the relationship between the condition of asthma (controlled or not) and the intensity of migraine attacks in 35 patients with asthma and migraine. We investigated the degree of changes in symptoms (change in fractional exhaled nitric oxide [FeNO], change in peak expiratory flow [PEF], and change in Asthma Control Test [ACT]) during exacerbation of asthma and observed the intensity of the headache (change in visual analog scale [∆VAS]). Next, we investigated the correlation between ∆VAS and biomarkers of bronchial asthma.

**Results:**

When migraine was aggravated, indexes of asthmatic symptoms (ACT, PEF, and FeNO) were also exacerbated. We found a linkage between insufficiently controlled asthma and aggravation of migraine. Moreover, the three indexes of asthma (ACT, PEF, and FeNO) reviewed in this study showed a close correlation. There was a positive correlation between the degree of exacerbation of migraine symptoms and the degree of change in the three indexes. In 14 of 35 patients, administration of a short-acting β2 agonist for asthma attacks indirectly relieved headache.

**Conclusions:**

here is a close correlation between the condition of asthma attacks and the intensity of migraine attacks.

## Introduction

Migraine can be accompanied by several comorbidities, that is, common background ailments existing not coincidentally but in association with migraine. Cardiovascular diseases, depression, and allergic diseases including bronchial asthma are receiving attention as comorbidities of migraine ^(1), (2)^.

Many patients with migraine experience allergic diseases at the same time, suggesting comorbidity between these two diseases ^(3)^. Numerous reports have been presented regarding interrelations between headache and general respiratory disorders and/or allergic symptoms, some suggesting that allergic rhinitis cases have a higher rate of complications ^(4), (5)^. Case reports describe both child and adult patients ^(5), (6)^.

Comorbidity between migraine and bronchial asthma, a typical allergic disease, has been reported sporadically since 1920. Pasteur Vallery-Radot et al. reported that individuals who have migraine frequently experience accompanying allergic diseases. Recent reports from Martin et al. suggest that patients with asthma have more than double the risk of chronic migraine developing than that of individuals with no respiratory disorders ^(7)^. Berg et al. directly confirmed that tar allergy reproducibly triggered migraine ^(8)^.

It is difficult to explain all these comorbidities using conventional propositions on mechanism of occurrence, such as migraine blood vessel theory, cortical spreading depression theory, or trigeminovascular theory ^(9)^. Analysis of these comorbidity cases is essential to be able to gain a thorough understanding of the pathophysiological mechanism of migraine.

This study reviews comorbidity between migraine and allergy and/or bronchial asthma to investigate whether exacerbation of these conditions is correlated, and what features such correlation might show.

## Materials and Methods

This study observed 35 patients with bronchial asthma with migraine (average age 35.1 ± 13.3 years [mean ± standard deviation (SD)], aged from 14 to 73 years, male-to-female ratio [5: 30]) who regularly visited the Allergy Center of our hospital for a year. We checked their asthma diary to confirm descriptions indicating exacerbation of migraine during insufficient asthma control (at the time of attacks) when they visited for regular consultations or appointments.

The complication of allergic rhinitis was found in 32 patients (Table 1). All patients had immunoglobulin E antibodies against life environment allergens that caused bronchial asthma. The severity of their asthma, based on the Asthma Prevention and Management Guideline published by the Japanese Society of Allergology ^(10)^, was 14 severely persistent and 20 moderately persistent. All 35 patients had been prescribed inhaled steroids as a basic treatment, and short-acting β2 agonist (SABA) and leukotriene receptor antagonist as additional therapy. Seven patients had received sustained-release theophylline.

**Table 1.** Background of all patients.

			headache characteristics						
No	Age	Sex	Pain(part of head)	Duration	Characteristics	Accompanying symptoms	Aura	Asthma severity	Comorbidity
1	42	F	occipital	up to 24 hrs	stabbing	nausea	scintillation	severe	AR, DA, AIA
2	25	F	both sides	up to 12 hrs	pulsating	(-)	scintillation	mild	AR, DA
3	36	F	both sides	2-3 days	pulsating	photophobia, nausea	scintillation	severe	AR, AC, AD
4	19	F	forhead	up to 24 hrs	stabbing	nausea	(-)	severe	AR, DA
5	31	F	rt side	4-6 hrs	pulsating	nausea	(-)	severe	AR
6	59	F	occipital	4-6 hrs	pulsating	nausea	(-)	severe	AR, AC
7	41	F	both sides	4-6 hrs	stabbing	nausea	(-)	moderate	AR
8	33	F	both sides	up to 6 hrs	pulsating	nausea	scintillation	moderate	AR, PA
9	33	F	forhead	up to 24 hrs	pulsating	nausea	scintillation	severe	AR, PA
10	33	F	both sides	up to 24 hrs	pulsating	(-)	scintillation	severe	AR
11	40	F	rt side	up to 24 hrs	pulsating	nausea	(-)	moderate	AR
12	64	M	forehead	up to 24 hrs	pulsating	photophobia, nausea	scintillation	severe	AR
13	73	M	lt side	up to 24 hrs	pulsating	nausea	(-)	severe	AR
14	43	M	rt side	up to 24 hrs	pulsating	(-)	scintillation	moderate	FA
15	42	F	both sides	4-6 hrs	pulsating	nausea	(-)	severe	AR
16	45	M	both sides	up to 24 hrs	pulsating	(-)	scintillation	severe	AR
17	32	F	forehead	4-6 hrs	pulsating	nausea	scintillation	moderate	AR・FA
18	27	F	both sides	up to 12 hrs	pulsating	nausea	scintillation	moderate	AR
19	22	F	forehead	up to 12 hrs	pulsating	nausea	scintillation	moderate	AR
20	24	F	forehead	up to 6 hrs	pulsating	nausea	(-)	moderate	AR
21	20	F	both sides	up to 6 hrs	pulsating	nausea	(-)	moderate	AR, PA
22	35	F	forehead	up to 6 hrs	pulsating	nausea	(-)	moderate	AR
23	34	F	both sides	up to 24 hrs	pulsating	nausea	(-)	moderate	AR
24	17	F	both sides	up to 24 hrs	pulsating	photophobia	(-)	moderate	AR
25	14	M	both sides	up to 24 hrs	pulsating	phonophobia, photophobia	(-)	moderate	AR, AC
26	41	F	lt side	4-6 hrs	pulsating	phonophobia, photophobia	(-)	moderate	AR, AC
27	21	F	both sides	up to 24 hrs	pulsating	photophobia	scintillation	moderate	AR, AC
28	42	F	top	up to 6 hrs	pulsating	(-)	scintillation	severe	AR
29	45	F	both sides	up to 6 hrs	pulsating	photophobia	(-)	moderate	AR
30	26	F	forehead	up to 6 hrs	pulsating	photophobia, nausea	scintillation	severe	AD, AD
31	33	F	forhead+both sides	up to 6 hrs	pulsating+stabbing	photophobia	(-)	moderate	AR, AC
32	52	F	forhead+both sides	up to 24 hrs	pulsating	(-)	scintillation	moderate	AR
33	19	F	forhead+both sides	up to 6 hrs	pulsating+stabbing	photophobia, nausea	scintillation	moderate	AR
34	39	F	both sides	up to 12 hrs	pulsating	photophobia, nausea	scintillation	severe	AR
35	26	F	forehead	up to 6 hrs	pulsating	photophobia, nausea	scintillation	moderate	AR

AD: atopic dermatitis; AIA: aspirin induce asthma; AC: allergic conjunctivitis, AR: allergic rhinitis; DA: drug allergy; F: female; FA: food allergy; lt: left; M: male; PA: pediatric asthma; rt: right. Age, sex, location of pain, duration of headache, characteristics, collateral symptoms, aura, severity of asthma, and other complications are included.

We conducted a medical chart-based retrospective assessment of elements later described among those patients at the time of their visits.

To diagnose migraine, we used the diagnosis criteria from the International Classification of Headache Disorders, Third Edition ^(11)^; 24 cases were diagnosed as migraine without aura and 11 cases as migraine with aura. The asthma control status was checked daily using an asthma diary. Simultaneously, the characteristics, location, intensity (visual analog scale [VAS]), duration, and interval of headache attacks were recorded.

To diagnose bronchial asthma, we followed the Global Initiative for Asthma ^(12)^ diagnostic criteria and the Asthma Prevention and Management Guideline 2018 published by the Japanese Society of Allergology (Asthma Prevention and Management Guideline 2018 Compilation Committee, Asthma Guideline Team, the Japanese Society of Allergology). We evaluated the levels of asthma treatment and asthma control using a questionnaire called the Asthma Control Test (ACT) ^(13)^, along with fractional exhaled nitric oxide (FeNO) ^(14)^, an indicator of allergic inflammation of the air passages. FeNO measurements were conducted using NIOX MINO (Astocrine) at an expiratory flow rate of 50 mL/s and intraoral pressure of 5-20 cmH_2_O (the test was conducted once) ^(15)^.

To measure the degree of airway narrowing, we also asked patients to perform peak expiratory flow (PEF) monitoring ^(16)^ every morning and night, three times each, and to record the highest value of each. We conducted a statistical examination of the results using the paired *t-*test for FeNO and PEF, and the Wilcoxon signed-rank test for VAS and ACT. This study received ethical approval from the Institutional Review Board of Saitama Medical University Hospital (approved numbers:14-119) and was registered in the University Hospital Medical Information Network (UMIN) Clinical Trials Registry (UMIN; 000012191).

Regarding the publication of this study, we have posted a notice on our hospital website using an opt-out format, and we have confirmed the explanation and consent of outpatients and recorded it in their medical records.

## Results

### Migraine attacks are more likely to occur when bronchial asthma is insufficiently controlled

**Figure 1.**
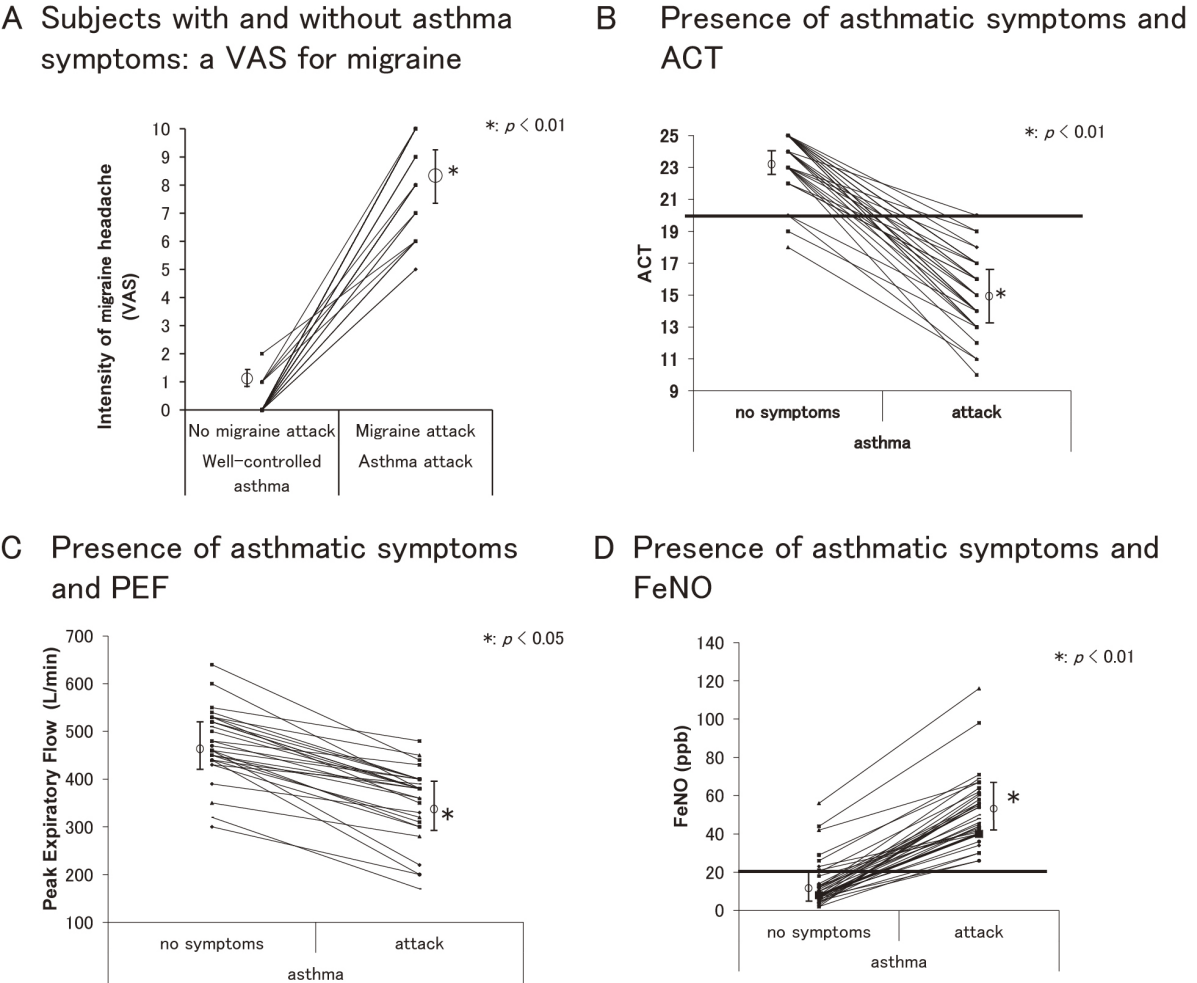
Figure 1A. Subjects with and without asthma symptoms: a VAS for migraine. Patients were examined for their degree of exacerbation (pain) of migraine using a VAS. We confirmed the presence of asthma symptoms (subjective symptoms, such as shortness of breath and asthma attacks) on the basis of their asthma diary or through consultations to study any changes. All patients who visited us without making an appointment were experiencing asthma attacks. At times of no migraine attacks, their asthma control was working sufficiently (no asthma attacks), and their migraine attacks corresponded to insufficient migraine control (with asthma symptoms/attacks). We conducted statistical verification of the results using the Wilcoxon signed-ranks test. Figure 1B. Presence of asthmatic symptoms and FeNO. To evaluate the level of allergic inflammation control of the airways, we measured the FeNO of 35 patients when they visited our center as outpatients or at the time of lack of asthma control. The cut-off lines of asthmatic inflammation of the airways are shown as a ruled line ^(32)^. The results were statistically reviewed using the paired *t*-test. Figure 1C. Presence of asthmatic symptoms and PEF monitoring. We recorded the PEF values at the time of lack of asthma control (during symptoms) in patients who had originally performed PEF monitoring to evaluate their asthma control. The results were statistically reviewed using the paired *t-*test. Figure 1D. Presence of asthmatic symptoms and ACT. With the aim of evaluating the level of asthma control, we conducted ACTs in 35 patients when they visited our center as outpatients or at times of lack of asthma control. Patients were also asked to record the intensity of headache. The border between sufficient and insufficient asthmatic control is shown as a ruled line ^(33)^. The results were statistically reviewed using the Wilcoxon signed-ranks test. ACT: Asthma Control Test; FeNO: fractional exhaled nitric oxide; PEF: peak expiratory flow; VAS: visual analog scale.

Throughout the one-year observation period, we confirmed that migraine occurred when asthma control was not sufficiently effective (at the time of asthma attacks/symptoms). Headache intensity (VAS) increased significantly during migraine attacks, from 0.2 ± 0.5 at times of no attacks to 8.3 ± 1.7 (mean ± SD) during attacks (Figure 1A). ACT scores during insufficient asthma control were 15.0 ± 2.4, which were significantly lower, showing exacerbation, than the ACT scores of 23.4 ± 1.8 during well-controlled periods (Figure 1B). We studied the PEF of 30 patients who experienced severe asthma, who had conducted PEF at home to record the state of their asthma control. The value of PEF during insufficient asthma control was 351.7 ± 76.3 L/min, significantly lower than that during well-controlled periods at 473.0 ± 73.7 L/min (Figure 1C). Changes in the migraine intensity are presented in Figure 1. We also found increased FeNO, which indicates the degree of allergic inflammation of the airways, during times of insufficient asthma control (before exacerbation 13.8 ± 12.5 parts per billion [ppb]; during exacerbation 52.7 ± 18.4 ppb) (Figure 1D).

### Migraine VAS corresponds to changes in asthma condition

**Figure 2.**
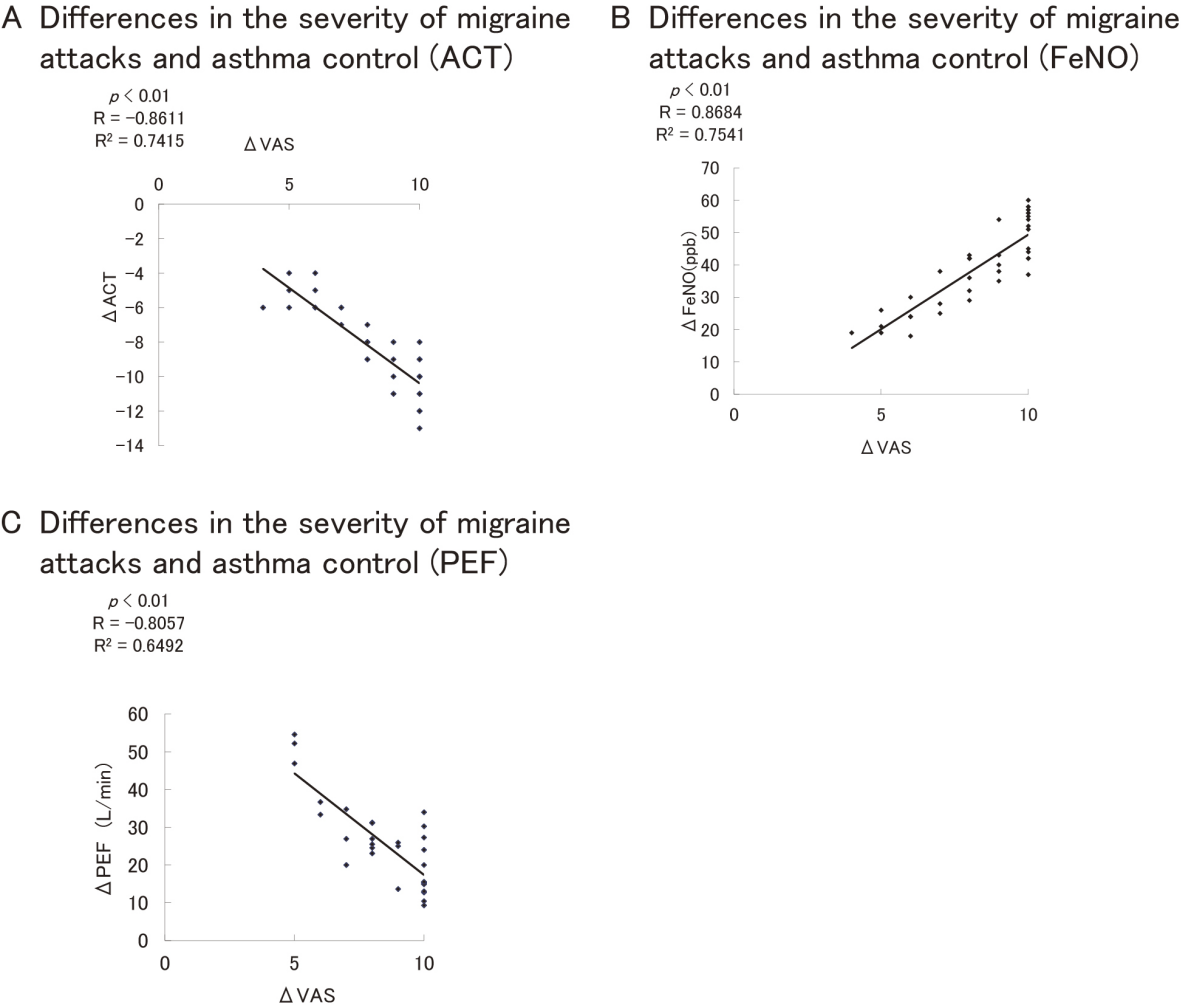
Correlation between VAS of migraine and bronchial asthma. A correlation is seen in the difference in intensity of migraine and control markers of bronchial asthma (ACT [Figure 2A], FeNO [Figure 2B], and PEF [Figure 2C]) between times of sufficient control and insufficient control. Figure 2A. Differences in the severity of migraine attacks and asthma control (ACT). We evaluated the relationship between the severity of headache during migraine attacks (VAS) and asthma symptom assessment and the differences between the severity of headache and ACT during nonmigraine attacks. Figure 2B. Differences in the severity of migraine attacks and asthma control (FeNO) We investigated the association between the severity of headache during migraine attacks (VAS) and the severity of asthma airway inflammation (FeNO), and the differences between these measures during migraine-free periods. Figure 2C. Differences in the severity of migraine attacks and asthma control (PEF). We evaluated the association between the severity of headache during migraine attacks (VAS) and the severity of asthma (PEF), and the differences between the severity of headache and PEF during nonmigraine attacks. ACT: Asthma Control Test; FeNO; fractional exhaled nitric oxide; PEF: peak expiratory flow; VAS: visual analog scale.

The relationship between intensity of migraine headache (change in VAS [∆VAS]) and the values for change in ACT (∆ACT) scores showed a significantly negative correlation (Figure 2A: p < 0.01, R = −0.8611, R^2^ = 0.7415). The relationship between intensity of migraine headache (∆VAS) and the values for change in FeNO (∆FeNO) also showed a significantly positive correlation (Figure 2B: *p* < 0.01, R = 0.8684, R^2^ = 0.7541). Conversely, the values for ∆VAS and change in PEF (∆PEF) had a significantly negative correlation (Figure 2C: p < 0.01, R = −0.8057, R^2^ = 0.6492). These results indicate migraine intensity to be correlated with poor control of asthma. In addition, the intensity of migraine was weaker, and frequency of migraine attacks was reduced, when asthma symptoms were relieved (data not shown).

### Migraine attacks during asthma attacks can be alleviated by β-agonists.

Symptoms of migraine attacks were originally controlled by medication, including on-demand use of nonsteroidal anti-inflammatory drugs. During the study, the use of a SABA as an acute treatment for asthma attacks caused indirect alleviation of migraine headache in 14 of 35 patients.

## Discussion

Our study examined the relationship between bronchial asthma and migraine through a retrospective review of medical charts to investigate ways the symptoms of migraine vary as a function of changes in asthma control. We found that when migraine (intensity of headache: VAS) was aggravated, indexes of asthmatic symptoms (ACT, PEF, and FeNO) were similarly exacerbated. In other words, there appears to be a linkage between insufficient asthma control and aggravation of migraine. We also studied the correlation between VAS and control markers of bronchial asthma. All three indexes reviewed in this study showed a correlation, indicating a causal relationship between migraine attacks and asthma control, in addition to the presence of common pathological symptoms.

To explain the reason migraine symptoms are interrelated with the mechanism of allergy, Churshmann et al. postulated an unstable autonomic nervous response to allergy, although accepting the sympathicovascular theory and serous meningitis theory, which argues that vasospasm and effusion of serous fluid are final common pathways causing migraine ^(17)^. The results of Hahn et al., obtained from animal testing in which vapospasm in frogs was induced by allergic responses, also indicated that patients with allergy can show vasospasm as a protective response to prevent allergens from spreading to the entire body ^(18)^. In English-speaking countries, bronchial asthma is referred to as “pulmonary migraine,” ^(19)^ and the interrelation between migraine and asthma was reported in 1952 ^(20)^. There have also been reports on histamine ^(21)^, a chemical mediator that causes allergic symptoms, including asthma, which can cause the vasculature to induce migraine ^(22)^; neurological behavior of leukotriene D4 in the central airways ^(23)^; and receptor antagonists against leukotrienes (leukotriene modifiers) leading to relief of migraine symptoms ^(24)^. Martin et al. stated that migraine and asthma are both associated with the inflammation/activation of smooth muscles in the vascular system or airways, and therefore, asthma-related inflammation can cause exacerbation of migraine ^(7)^. It is reported that patients with migraine show an increased risk of bronchial asthma developing, with a complication rate of 40% and a relative risk of 1.59 (95% confidence interval 1.54-1.65) ^(25)^. However, there have been no reports on changes in migraine symptoms when asthma is controlled. In this study, we observed the interrelationship between aggravation of asthma and migraine, although the number of patients involved was small owing to the difficulty of finding patients with both conditions. Our results suggest that the concentrations of chemical mediators, which induce asthma symptoms, increase during asthma attacks, affecting the cerebral vasculature and the neurological functions of the airways in ways that trigger migraine.

It is clear from past reports and the results of this study that there is an association between migraine and bronchial asthma. Our results do not reveal the mechanism by which asthma and migraine affect each other. However, we speculate the possible involvement of nitrous oxide (NO), a biomarker of allergic inflammation of the airways, as an underlying symptom of asthma. There have been numerous reports on NO in relation to the mechanism of migraine ^(26), (27)^. In vitro examinations assuming allergy ^(28)^, axon reflex theory regarding asthma ^(29)^, and the role of NO in axon reflex ^(30)^ have also been reported. We have observed that the ability of the vascular endothelium to produce NO increases during migraine attacks ^(31)^, but the mechanism by which NO induces migraine attacks has not yet been clarified. Because NO is metabolized to NO_2_^-^ and NO_3_^-^ in the blood, it is not possible to directly link NO levels in the blood to exhaled NO, especially given exhaled NO also appears to be produced from the respiratory epithelium ^(32)^. The results of this study suggest high NO production in the respiratory epithelium of patients with migraine. We believe it is necessary to extend this study using a larger number of cases.

This study has a limitation. Patients with well-controlled asthma and no migraine attacks, and those with uncontrolled asthma and migraine attacks, were included. However, to firmly establish the association between these conditions, data on asthma exacerbation without a migraine attack and migraine attacks without asthma exacerbation are also needed.

In conclusion, this study has shown an association between asthma attacks and migraine pain attacks, suggesting a relationship. The exact pathology is not known. Many patients experience both diseases. The provision of comprehensive patient care necessitates the collaboration of various specialists or the total management by general practitioners.

We hope this study will raise awareness among physicians and improve patient satisfaction.

## Article Information

### Acknowledgments

Hidetomo Nakamoto provided invaluable support as the head of the medical practice when this research was conducted. He died before the submission of the study, so he was not included as a co-author. We express our sincere gratitude to him.

### Author Contributions

Takehito Kobayashi and Nobuo Araki designed the study, were responsible for the conventional methods, and performed the experiments; Takehito Kobayashi, Nobuo Araki, Tomoko Suzuki, Makoto Nagata, and Naotoshi Tamura analyzed the data; and Takehito Kobayashi, Nobuo Araki, Takao Atsumi, Yotaro Takaku, Keiji Yamamoto, and Naotoshi Tamura wrote the manuscript.

### Conflicts of Interest

None

### Institutional Review Board Approval Code and Name of the Institution

This study was approved by the local Ethics Committee of Saitama Medical University Hospital (approval numbers:14-119) and was registered in the UMIN Clinical Trials Registry (UMIN; 000012191).
